# Proteomic Analysis of Tissue from α1,3-galactosyltransferase Knockout Mice Reveals That a Wide Variety of Proteins and Protein Fragments Change Expression Level

**DOI:** 10.1371/journal.pone.0080600

**Published:** 2013-11-14

**Authors:** Louise Thorlacius-Ussing, Maja Ludvigsen, Svend Kirkeby, Henrik Vorum, Bent Honoré

**Affiliations:** 1 Department of Ophthalmology, Aalborg University Hospital, Aalborg, Denmark; 2 Department of Biomedicine, Aarhus University, Aarhus, Denmark; 3 Institute of Odontology, University of Copenhagen, Copenhagen, Denmark; UNIFESP Federal University of São Paulo, Brazil

## Abstract

A barrier in a pig-to-man xenotransplantation is that the Galα1-3Galβ1-4GlcNAc-R carbohydrate (α-Gal epitope) expressed on pig endothelial cells reacts with naturally occurring antibodies in the recipient’s blood leading to rejection. Deletion of the α1,3-galactosyltransferase gene prevents the synthesis of the α-Gal epitope. Therefore, knockout models of the α1,3-galactosyltransferase gene are widely used to study xenotransplantation. We have performed proteomic studies on liver and pancreas tissues from wild type and α1,3-galactosyltransferase gene knockout mice. The tissues were analyzed by two-dimensional polyacrylamide gel electrophoresis and liquid chromatography - tandem mass spectrometry. The analyses revealed that a wide variety of proteins and protein fragments are differentially expressed suggesting that knockout of the α1,3-galactosyltransferase gene affects the expression of several other genes.

## Introduction

The first barrier in a pig-to-man xenotransplantation is that the Galα1-3Galβ1-4GlcNAc-R carbohydrate (α-Gal epitope) expressed on pig endothelial cells will react with naturally occurring xenoreactive circulating antibodies in the recipient´s blood leading to hyperacute rejection of the discordant xenograft within a few minutes. Deletion of the α1,3-galactosyltransferase gene will prevent synthesis of the α-Gal epitope and generation of α-Gal transferase knockout animals is therefore an important step for xenotransplantation of vascularised organs [1]. The results from grafting α-Gal transferase knockout organs into non-human primates show that many biological barriers such as human antibodies against pig antigens must be overcome before being a clinical reality [2]. Thus, carbohydrate antigens other than the Gal antigen (i.e. non-Gal antigens) such as blood group AO and related antiges, Tn and Sialyl-Tn antigens and perhaps other saccharides to which humans have preformed antibodies may cause pig xeno-rejection [3,4]. Further, knockout of a gene may eliminate the construction of its specific gene product, but it may also influence the expression of other proteins that may be up- or downregulated [5,6]. The α-Gal epitope is expressed in wild type mice and therefore the α1,3-galactosyltransferase knockout models have been extensively used in xenotransplantation studies. To determine if other proteins are differentially expressed in α1,3-galactosyltransferase knockout mice compared to wild type mice, we performed proteomic analyses of liver and pancreas tissues by using two dimensional polyacrylamide gel electrophoresis (2D-PAGE) and liquid chromatography - tandem mass spectrometry (LC-MS/MS) to identify such protein candidates. 

## Materials and Methods

C57BL/6 mice, 3-months-old, all male were included in the study. All procedures were conducted under protocols approved by the Danish Animal Care and Ethics Committee and conducted in accordance with the Danish Animal Experimentation Act and European Convention for the protection of vertebrate animals used for experimental and other scientific purposes. Half of the mice were α-Gal knockout mice, lacking a functional α1,3-galactosyltransferase gene, C57BL/6/CimlKvl-Tgaltm1Tea. The mice were generated as described by Tearle et al. [7] hereafter termed KO mice. The phenotype was confirmed by development of cataracts at 4–6 weeks of age and by PCR. C57BL/6JBomTac mice, hereafter termed wt mice, were used as controls. The mice had free access to food and water and were sacrificed between 10 a.m. and noon. Specimens from the liver and pancreas were frozen in isopentane cooled to -150° C with liquid nitrogen. Some samples were cut on a cryostat in 6µ sections for histochemisty while other were processed for electrophoresis. The lectin to detect the α-Gal epitope in tissue sections was a biotinylated *Griffonia simplicifolia* isolectin (GS1-B4; EY Laboratories, San Mateo, CA, USA). The sections were incubated for 24 h at 4° C with 5 μg/ml of the lectin, diluted 1:200 from a stock solution of 1 mg/ml in TBS. The incubation medium contained 20 mM CaCl_2_ and MgCl_2_. After a 3x5 min rinse in TBS the sections were immersed in Alexa Fluor 488 streptavidin conjugate for 30 min. The sections were mounted with a fluorescence mounting medium with the DNA binding agent 4´-6-diamidino-2-phenylindole (DAPI; Vector Lab, Burlingame CA, USA).

### Two-dimensional gel electrophoresis (2D-PAGE)

Liver and pancreas tissue samples were taken from three wt and three KO mice. Tissue samples were dissolved in lysis buffer consisting of 9 M urea, 2% DTT, 2% Triton X-100 and 2 % IPG buffer. Horizontal isoelectric focusing was performed using a non-linear pH3-10NL IPG strip, rehydrated for 20 hours at room temperature with a rehydration buffer (8 M urea, 2 % CHAPS, 2% IPG-buffer, 0,3% DDT). The first dimension was carried out on a Multiphor ΙΙ Electrophoresis unit at 500 V for 5 hours and at 3500 V for 14.5 hours. Prior to the second dimension, the IPG strip was equilibrated twice with an equilibration buffer (0.05 M Tris-base, 6 M Urea, 26% glycerol, 1 % SDS), and transferred to a polyacrylamide gel. The second dimension separation was run vertically at 50 V for 19 hours [8]. One gel was performed for each tissue sample from one mouse, thus three biological replicates were analysed for each tissue.

### Silver staining

Visualisation was achieved by sliver staining, suitable for quantitative protein analysis [8,9]. Briefly, gels where fixed overnight in a fixation solution (50% ethanol, 12 % acetic acid and 0.0185% formaldehyde), then washed three times for 20 minutes in 35% ethanol, pre-treated for one min in 0.02 % Na_2_S_2_O_3_, and rinsed in water 3 times for 2-3 min. Staining of gels with silver nitrate was preformed for 20 minuets, after which they again where rinsed twice with water. Gels where developed using a developer solution (6% Na_2_CO_3_, 0.0185% formaldehyde, 0.0004% Na_2_S_2_O_3_, 5 H_2_O) for approximately 3 min. Development where finally stopped in a stop solution (40% ethanol, 12% acetic acid). The gels where dried in cellophane sheets and sealed in plastic bags.

### Image analysis

Silver stained gels where scanned using an ImageQuant LAS-4000 (GE Healthcare) and TIFF images of gels where imported into the PDQuest software analysis program. Two proteomic analyses where done; one with pancreatic tissue and one with hepatic tissue. The 2D-gels where divided in two groups; one representing wt mice and another representing KO mice. Protein spots where automatically defined and adjusted to the background. The quality of each protein spot was critically evaluated in order to ensure that all relevant protein spots were identified. The pixel-intensity of each protein spot was translated to a proportional protein volume, which was normalized to the total density of the gel and given in parts per million, ppm. Finally, re-analysis was done manually on all matched spots. Differentially expressed spots were defined as spots that differed at least 2-fold in average relative volume between the groups of wt and KO mice. The results where considered to be significant when p<0.05 using a students t-test. These spots where selected for further identification by LC-MS/MS.

### Protein identification

Protein identification by LC-MS/MS was performed essentially as previously described [8]. Briefly, gels containing protein spots selected for identification were re-hydrated in water, the cellophane sheets were peeled off and the protein spots were excised from the gels. Proteins were *in-gel* digested with trypsin. Gel pieces were first dehydrated in acetonitrile, then dried and the proteins reduced for 1h at 56°C in 10 mM dithiotreitol (DTT) and 100 mM NH_4_HCO_3_. The solution was exchanged with 55 mM iodoacetamide in 100 mM NH_4_HCO_3_ for 45 min. Then the gel pieces were washed in 100 mM NH_4_HCO_3_, dehydrated in acetonitrile, rehydrated in 100 mM NH_4_HCO_3_, dehydrated in acetonitrile, dried and swelled in digestion buffer (50 mM NH_4_HCO_3_, 5 mM CaCl_2_ and 12.5 ng/μl trypsin Gold (mass spectrometry grade; Promega, Madison, WI, USA). Digestion was performed overnight at 37°C and the peptides were extracted by 1 change of 20 mM NH_4_HCO_3_ and 3 changes of 5% formic acid in 50% acetonitrile. The samples were finally dried and the peptides resuspended in 6 μl of buffer A (water/acetonitrile/formic acid, 97.7/2/0.3, V/V/V). The peptides were separated using an inert nano LC system composed of a Famos micro autosampler, a Switchos micro column switching module and an Ultimate micro pump from LC Packings (San Francisco, CA) before MS analysis. Of the *in-gel* digested samples 5 μl was preconcentrated and desalted on a 300 μm inner diameter x 5 mm nano-precolumn (LC Packings) packed with 5 μm C18 PepMap100 material. A 75 μm inner diameter x 15 cm nano-column packed with 3 μm C18 PepMap100 material was used to separate the peptides. Elution from the column was made with a gradient by mixing decreasing volumes of buffer A with increasing volumes of buffer B (water/acetonitrile/formic acid, 9.7/90/0.3, V/V/V). The peptides were eluted into the nano electrospray ion source of the quadrupole time-of-flight (Q-TOF) Premier mass spectrometer (Waters). MS survey scans were acquired using MassLynx 4 SP4 (Waters) from m/z values between 450-1500. The instrument was operated in a data-dependent MS to MS/MS switching mode. Doubly, triply and quadruply charged peptide ions detected in MS survey scans triggered a switch to MS/MS for obtaining peptide fragmentation spectra with an interval of *m/z* values between 50-1800. [Glu^1^]-fibrinopeptide B (GFP), 300 fmol/μl, was used as lock mass injected at a flow rate of 300 nl/min. GFP was also used to calibrate the TOF unit. Raw data were processed using ProteinLynx GlobalServer 2.1 (Waters) with processing parameters: Background Subtract: Normal, Background Threshold: 35%, Background Polynomial: 5, Smoothing Type: Savitzky-Golay, Smoothing Iterations: 2, Smoothing Window: 2 channels, Deisotoping Type: Normal, Deisotoping Threshold: 1%. The processed data were used to search the mouse fraction of the Swiss-Prot database (release 2011_11 or 2013_05) using the on-line version of the Mascot MS/MS Ion Search facility (Matrix Science, Ltd., http://www.matrixscience.com) [10]. Searching was performed with doubly, triply and quadruply charged ions with up to 2 missed cleavages, a peptide tolerance of 20 ppm, one variable modification, Carbamidomethyl-C, and an MS/MS tolerance of 0.05 Da. Spectra of dubious identifications were evaluated manually and omitted if they were of insufficient quality. Contaminating peptides and cross-contaminating peptides from previous samples including keratins, trypsin, BSA and casein were disregarded. At least one ‘bold red’ peptide with scores giving a less than 5% probability that the observed match was a random event was required in the search for protein hits. All peptides for these hits are reported. If the first search in the mouse fraction of the Swiss-Prot database did not give any hit an additional search in the whole database was performed.

## Results

**Figure 1:**
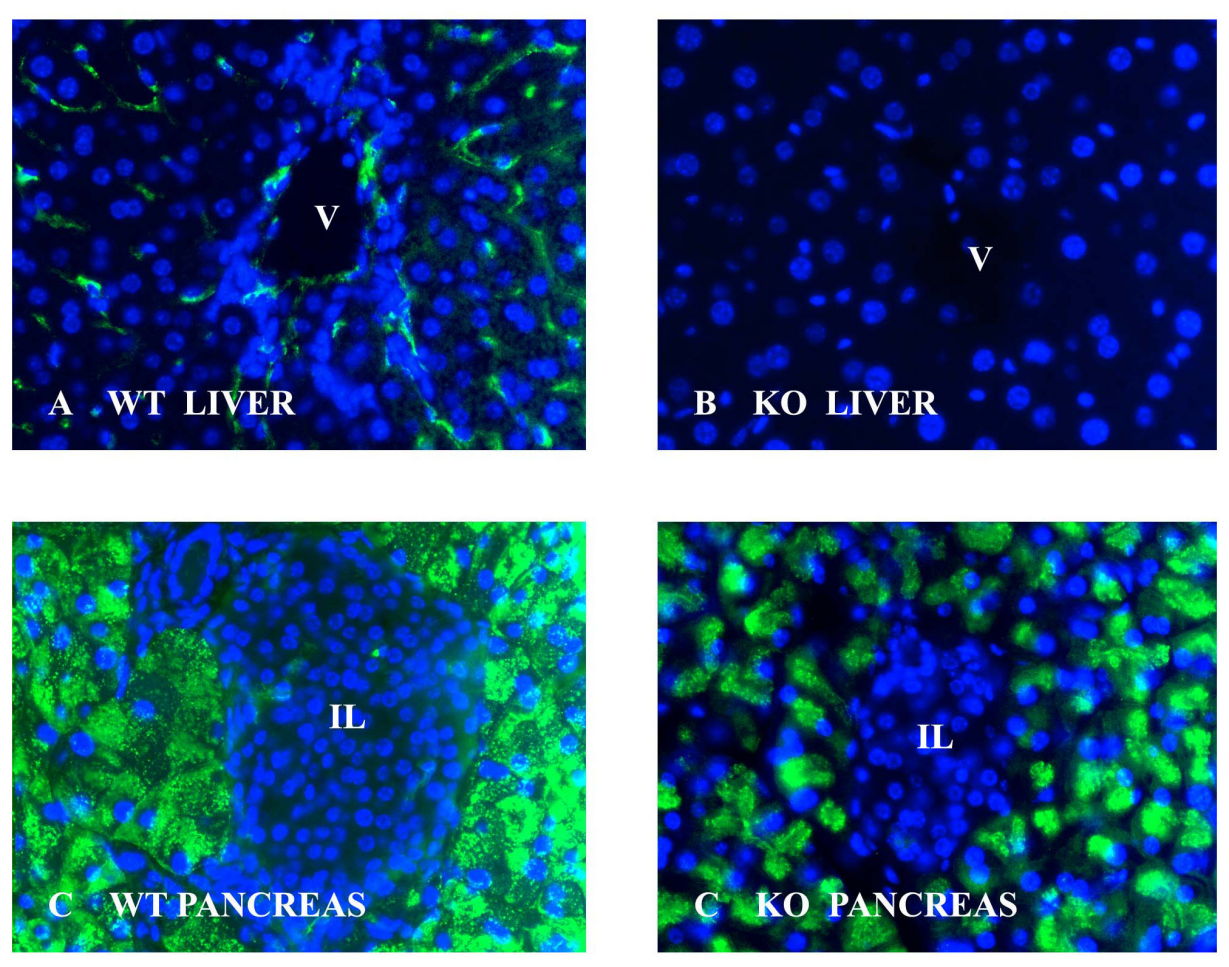
GS1B4 staining of wt and α1,3-galactosyltransferase KO mouse tissues. The lectin stains the central vein (v) and the sinusoids are outlined in sections from the wt mouse liver (A) while there is no reaction in sections from the KO mouse liver (B). There is no staining of the blood vessels in pancreas from neither the wt mouse (C) nor from the KO mouse (D). The endocrine cells in islets of Langerhans (IL) are unstained while the exocrine cells in both the wt and the KO mouse show reaction. The green fluorescence reflects lectin staining and blue fluorescence reflects DAPI staining of the nuclei.

**Figure 2:**
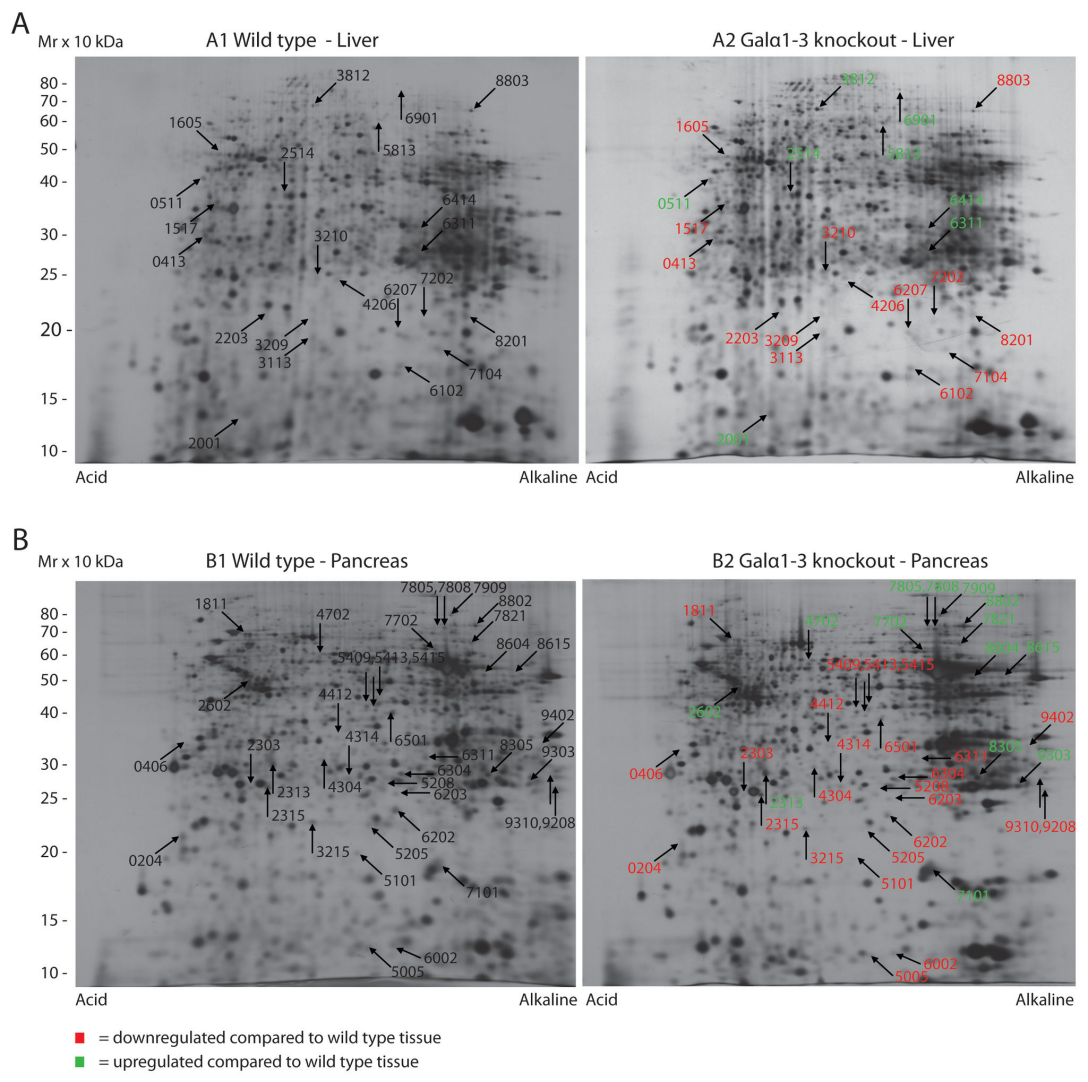
2D-PAGE images of wt and α1.3-galactosyltransferase KO mouse tissues. Liver (A) and panacreas (B). Proteins which are at least 2-fold differentially expressed are marked with red (downregulated) and gren (upregulated). Identified proteins are listed in Tables 1 and 2.

The staining pattern of the tissues used for the present analysis is shown in Figure 1. As expected GS1B4 stains the endothelial cells in the central vein as well as in the sinusoids of the wt liver whereas the α1,3-galactosyltransferase KO liver is unstained. In the pancreas the endothelial cells are unstained while the exocrine cells are stained in the mouse wt pancreas as well as in the KO pancreas. Figure 2 shows representative 2D-gels of wt and KO tissues from mouse liver and pancreas, respectively. Spots whose expression differed by at least 2-fold between the wt and the KO tissues are highlighted.

### Liver

**Figure 3:**
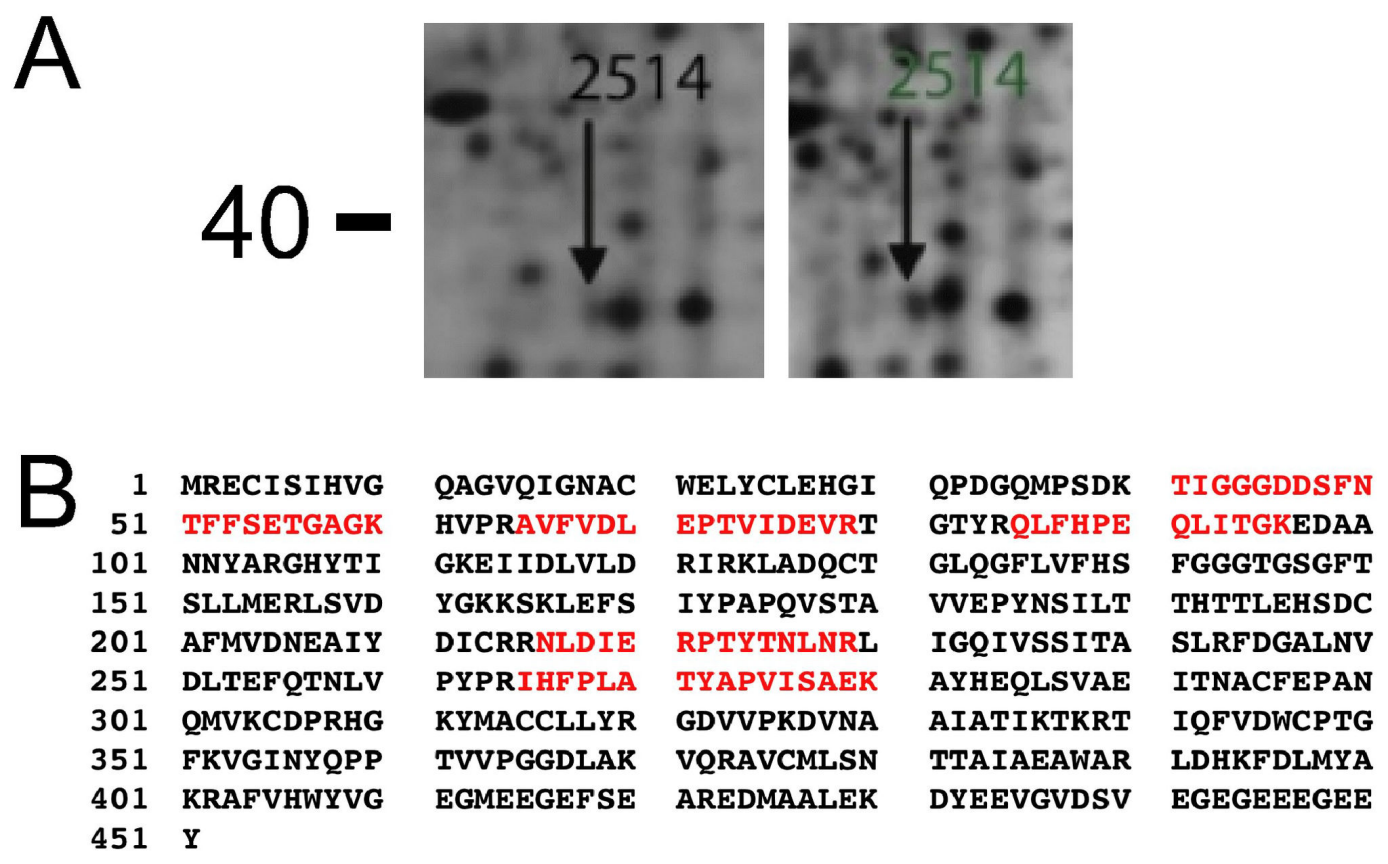
Spot No. 2514 is a fragment of α-tubulin A) Spot 2514 migrates with a molecular mass below 40 kDa. The identified peptides from the spot are distributed in the N-terminal of α-tubulin and spot 2514 thus corresponds to a fragment of α-tubulin, since α-tubulin possesses a molecular mass around 50 kDa.

In total 22 spots were found to be differentially expressed in α1,3-galactosyltransferase gene KO mice relative to wt mice as shown in Figure 2 panel A. Eight spots were upregulated, marked with green numbers, and 14 spots where downregulated, illustrated by red numbers. All spots were excised and subjected to mass spectrometry identification as summarised in Table 1. Eighteen spots were successfully identified. Of these, 12 were found to contain one protein that accounts for the observed change in expression. Six spots contained more than one protein (Nos. 0511, 2514, 3113, 6207, 6311 and 7104) in which case, we cannot with certainty conclude which of them accounts for the observed change in expression level. Interestingly, in the vast majority of cases we observed a major deviation of the observed molecular mass by 2D-PAGE with the theoretical molecular mass of the proteins, as illustrated in Figure 3. α-tubulin has a molecular mass around 50 kDa but spot 2514 migrated with a molecular mass below 40 kDa (Figure 3A). A map of the identified peptides showed that spot 2514 contained an N-terminal fragment of tubulin. A change in the concentration of a protein fragment may occur as a result of a change in the synthesis as well as a change in the degradation as it is the case with a full-length protein. Thus, it seems that for some proteins there is a substantial change in protein turnover in the KO liver. 

**Table 1:** Differentially expressed protein spots in mouse α1,3-galactosyltransferase KO liver.

**Spot**	**Ratio (KO/wt)**	**wt, Vol. ± SD (ppm)**	**KO, Vol. ± SD (ppm)**	**Peptides**	**Identification**	**Mr (Da)**	**Mascot Protein Score**	**Protein name**
0413	0.31	745 ± 251	229 ± 98	FELTGIPPAPR	HSP7C_MOUSE	70827	112	Heat shock cognate 70 kDa protein 3, fragment
				SINPDEAVAYGAAVQAAILSGDK				
0511	3.31	55 ± 20	181 ± 48	ITITNDQNR	GRP78_MOUSE /	72377 /	33	78 kDa glucose-regulated protein, fragment
				VYEGERPLTK /				
				VLVGANFEEVAFDEK	PDIA1_MOUSE	57023	29	Protein disulfide-isomerase, fragment
1517	0.47	648 ± 164	307 ± 75	N.I.*				
1605	0.36	479 ± 79	171 ± 165	N.I.				
2001	2.35	866 ± 232	2035 ± 482	ALDIAENEMPGLMR	SAHH_MOUSE /	47657	42	Adenosylhomocysteinase, fragment
2203	0.20	765 ± 212	151 ± 177	PPYTIVYFPVR	GSTP1_MOUSE	23594	81	Glutathione S-transferase P 1
				FEDGDLTLYQSNAILR				
2514	2.73	344 ± 56	937 ± 365	QLFHPEQLITGK	TBA1A_MOUSE or	50104 /	269	Tubulin α-1A/1B/1C chain, fragment
				AVFVDLEPTVIDEVR	TBA1B_MOUSE or			
				NLDIERPTYTNLNR	TBA1C_MOUSE /			
				IHFPLATYAPVISAEK				
				TIGGGDDSFNTFFSETGAGK /				
				KYEATLEK	ALBU_MOUSE	68648	30	Serum albumin, fragment
				LGEYGFQNAILVR				
3113	0.31	1526 ± 441	471 ± 228	TAHIVLEDGTK /	CPSM_MOUSE/	164514	50	Carbamoyl-phosphate synthase [ammonia], mitochondrial, fragment
				EKVDLLFLGK	ETFB_MOUSE	27606	24	Electron transfer flavoprotein subunit β, fragment
				GIHVEIPGAQAESLGPLQVAR				
3209	0.14	704 ± 198	97 ± 59	VNEAACDIAR	BHMT1_MOUSE	44992	45	Betaine--homocysteine S methyltransferase 1, fragment
3210	0.26	481 ± 99	126 ± 123	VAEQTPLTALYVANLIK	ALDH2_MOUSE	56502	32	Aldehyde dehydrogenase, mitochondrial, fragment
3812	2.90	196 ± 90	569 ± 189	KYEATLEK	ALBU_MOUSE	68648	65	Serum albumin
				LSQTFPNADFAEITK				
4206	0.37	594 ± 118	218 ± 173	NLPIYSEEIVEMYK	MYH9_MOUSE	226232	29	Myosin-9, fragment
5813	2.17	204 ± 48	441 ± 120	TPEELQHSLR	HACL1_MOUSE	63619	45	2-hydroxyacyl-CoA lyase 1
6102	0.31	1613 ± 517	500 ± 285	LLYDLADQLHAAVGASR	ETFA_MOUSE	34988	118	Electron transfer flavoprotein subunit α, mitochondrial, fragment
6207	0.34	910 ± 258	310 ± 94	IGASTQAAQR	CES3_MOUSE /	61749	142	Carboxylesterase 3, fragment
				DGASEEETNLSK				
				ISENMIPVVAEK /				
				TIPIDGDFFSYTR	ALDH2_MOUSE	56502	28	Aldehyde dehydrogenase, mitochondrial, fragment
6311	6.34	390 ± 494	2475 ± 1042	TIILYDTNLPDVSAK /	KHK_MOUSE/	32730	96	Ketohexokinase
				LALLSLTTGGTAEMYTK /	NQO2_MOUSE/	26231	63	Ribosyldihydronicotinamide dehydrogenase [quinone]
				TAHIVLEDGTK /	CPSM_MOUSE /	164514	60	Carbamoyl-phosphate synthase [ammonia], mitochondrial, fragment
				FQEAPEEGR	FMO5_MOUSE /	59962	56	Dimethylaniline monooxygenase [N oxide-forming] 5, fragment
				ASIYQSVVINTSK				
				IAVIGAGASGLTCIK /				
				TATPQQAQEVHEK	TPIS_MOUSE	32171	29	Triosephosphate isomerase
6414	4.46	244 ± 68	1089 ± 498	HAYGDQYR	IDHC_MOUSE	46644	43	Isocitrate dehydrogenase [NADP] cytoplasmic, fragment
				LDNNTELSFFAK				
6901	2.92	41 ± 11	119 ± 34	N.I.				
7104	0.28	1343 ± 337	375 ± 344	DHGDLAFVDVPNDSSFQIVK /	ARGI1_MOUSE /	34786 /	71	Arginase-1, fragment
				IDPSAPLDK	ADHX_MOUSE	39522	50	Alcohol dehydrogenase class-3, fragment
				AGDTVIPLYIPQCGECK				
7202	0.13	1033 ± 405	132 ± 121	VMEETFSYLLGR	ARGI1_MOUSE	34786	66	Arginase-1, fragment
8201	0.26	1671 ± 109	431 ± 378	N.I.				
8803	0.46	498 ± 40	228 ± 150	VAIAVAINQAIASGK	HUTU_MOUSE	74543	82	Urocanate hydratase
				TPNLSPEEEQLALR				

Searches were performed in the Swiss-Prot mouse database.

* N.I.: not identified.

 Identified upregulated protein spots included adenosylhomocysteinase fragment (2001), serum albumin (3812), 2-hydroxyacyl-CoA lyase 1 (5813) and isocitrate dehydrogenase [NADP] cytoplasmic fragment (6414). Downregulated spots included heat shock cognate 70 kDa protein 3 fragment (0413), glutathione S-transferase P 1 (2203), betaine-homocysteine S methyltransferase 1 fragment (3209), aldehyd dehydrogenase fragment (3210), myosin-9 fragment (4206), electron transfer flavoprotein subunit α fragment (6102), arginase-1 fragment (7202) and urocanate hydratase (8803). Only one protein of all identified proteins in Table 1 contained a putative N-glycosylation site, namely carboxylesterase 3 (spot 6207). 

### Pancreas

In total 39 differentially expressed spots were detected by 2D-PAGE analysis (Figure 2, panel B). All spots were excised and resulted in identification of 17 spots as summarised in Table 2. All of these were found to contain a single identification and about a third of them contained fragments of the proteins. Thus, there is also a substantial change in protein turnover for some proteins in the KO pancreas tissue. Of upregulated proteins, the following eight were identified; albumin fragment (2602), tensin-3 fragment (7702), serotransferrin (7808), chymotrypsin-like elastase family member 1 (8305), NADH dehydrogenase [ubiquinone] flavoprotein 1 (8604), ATP synthase subunit alpha, mitochondrial (8615), aconitate hydratase, mitochondrial (8802) and 3-hydroayacyl-CoA dehydrogenase type 2 (9303). Identified downregulated proteins were 78 kDa-glucose-regulated protein (1811), apolipoprotein A-1 (2315), glutathione S-transferase P1 (3215), 40S ribosomal protein S12 (5005), proteasome subunit β type-3 (5208), serum albumin fragment (5413), myeloperoxidase fragment (5415), GTP-binding protein SAR1a (6202) and serum albumin fragment (6304). Of all identified proteins in Table 2, only four contained putative N-glycosylation sites, i.e., myeloperoxidase, serotransferrin, receptor-type tyrosine-protein phosphatase α and chymotrypsin-like elastase family member 1.

**Table 2:** Differentially expressed protein spots in mouse α1,3-galactosyltransferase KO pancreas.

**Spot**	**Ratio (KO/wt )**	**wt , Vol ± SD (ppm)**	**KO, Vol ± SD (ppm**)	**Peptides**	**Identification**	**Mr (Da)**	**Mascot Protein Score**	**Protein name**
0204	0.18	1527 ± 41	282 ± 217	N.I.*				
0406	0.28	216 ± 61	60 ± 48	N.I.				
1811	0.15	926 ± 261	138 ± 70	ITITNDQNR	GRP78_MOUSE	72377	244	78 kDa glucose-regulated protein
				SDIDEIVLVGGSTR				
				TFAPEEISAMVLTK				
				ITPSYVAFTPEGER				
				IINEPTAAAIAYGLDKR				
				SQIFSTASDNQPTVTIK				
2303	0.14	420 ± 199	59 ± 67	N.I.				
2313	3.12	1032 ± 198	3221 ± 1189	N.I.				
2315	0.16	1030 ± 353	161 ± 132	HSLMPMLETLK	APOA1_MOUSE	30569	142	Apolipoprotein A-I
				SNPTLNEYHTR				
				VAPLGAELQESAR				
				VQPYLDEFQKK				
2602	4.88	242 ± 140	1182 ± 504	LGEYGFQNAILVR	ALBU_MOUSE	68648	55	Serum albumin, fragment
3215	0.33	989 ± 173	322 ± 172	PPYTIVYFPVR	GSTP1_MOUSE	23594	78	Glutathione S-transferase P 1
				FEDGDLTLYQSNAILR				
4304	0.30	619 ± 156	187 ± 127	N.I.				
4314	0.27	695 ± 194	190 ± 151	N.I.				
4412	0.17	350 ± 159	60 ± 62	N.I.				
4702	3.50	126 ± 67	440 ± 44	N.I.				
5005**	0.20	1120 ± 485	228 ± 88	LVEALCAEHQINLIK	RS12_BOVIN	14505	97	40S ribosomal protein S12
5101	0.18	493 ± 168	89 ± 45	N.I.				
5205	0.14	531 ± 80	72 ± 48	N.I.				
5208	0.32	791 ± 277	254 ± 57	FGPYYTEPVIAGLDPK	PSB3_MOUSE	22949	43	Proteasome subunit β type-3
5409	0.10	742 ± 293	74 ± 112	N.I.				
5413	0.23	448 ± 103	104 ± 84	LSQTFPNADFAEITK	ALBU_MOUSE	68648	48	Serum albumin, fragment
5415	0.12	1319 ± 488	160 ± 64	TITGHCNNR	PERM_MOUSE	81130	25	Myeloperoxidase, fragment
6002	0.27	1336 ± 400	359 ± 167	N.I.				
6202**	0.25	1960 ± 152	495 ± 158	LVFLGLDNAGK	SAR1A_BOVIN	22395	66	GTP-binding protein SAR1a
				EIFGLYGQTTGK				
6203	0.31	527 ± 163	164 ± 20	N.I.				
6304	0.19	1797 ± 817	341 ± 115	KYEATLEK	ALBU_MOUSE	68648	344	Serum albumin, fragment
				TPVSEHVTK				
				CCTLPEDQR				
				APQVSTPTLVEAAR				
				LGEYGFQNAILVR				
				RPCFSALTVDETYVPK				
				AADKDTCFSTEGPNLVTR				
				CCAEANPPACYGTVLAEFQPLVEEPK				
6311	0.25	774 ± 205	193 ± 160	N.I.				
6501	0.18	1558 ± 347	273 ± 185	N.I.				
7101	7.43	622 ± 776	4621 ± 1848	N.I.				
7702	4.88	198 ± 207	969 ± 366	FPDYGKIELVFSATPEK	TENS3_MOUSE	155491	36	Tensin-3, fragment
7805	3.23	109 ± 45	351 ± 22	N.I.				
7808	3.25	181 ± 185	586 ± 139	GTDFQLNQLEGK	TRFE_MOUSE	76674	55	Serotransferrin
				LYLGHNYVTAIR				
7821	3.15	227 ± 32	717 ± 131	N.I.				
7909	5.37	73 ± 92	389 ± 89	N.I.				
8305	4.86	474 ± 578	2307 ± 238	NNVVAGYDIALLR	CELA1_MOUSE	28882	52	Chymotrypsin-like elastase family member 1
8604	3.11	227 ± 142	707 ± 130	GEFYNEASNLQVAIR	NDUV1_MOUSE	50802	138	NADH dehydrogenase [ubiquinone] flavoprotein 1, mitochondrial
				NACGSDYDFDVFVVR				
8615	3.55	178 ± 151	631 ± 181	HALIIYDDLSK	ATPA_MOUSE	59716	28	ATP synthase subunit α, mitochondria
8802	6.74	334 ± 138	2252 ± 282	NAVTQEFGPVPDTAR	ACON_MOUSE	85410	82	Aconitate hydratase, mitochondrial
9208	0.12	1380 ± 431	159 ± 200	N.I.				
9303	4.78	416 ± 340	1991 ± 219	VVTIAPGLFATPLLTTLPEK	HCD2_MOUSE	27402	89	3-hydroxyacyl-CoA dehydrogenase type-2
9310	0.24	1112 ± 165	272 ± 222	N.I.				
9402	0.20	607 ± 30	123 ± 153	N.I.				

Searches were performed in the Swiss-Prot mouse database. In case no protein hits were obtained a subsequent search was performed in all Swiss-Prot databases. These hits are indicated with **

* N.I.: not identified.

## Discussion

The α-Gal epitope may be stronger expressed in some organs than in other. As an example incubation with the α-galactose specific lectin GS1B4 stains the endothelial cells in the liver from wt mice [7] while there is no lectin reaction in the endothelial cells in the wt mouse pancreas [11]. It also seems that heterozygote GalT-KO pigs display a significantly lower activity for α1,3-galactosyltransferase in some organs than in other [12]. In the present analysis, we confirmed presence of the α-Gal epitope on the endothelial cells of the wt liver as well as the absence on the endothelial cells in the pancreas (Figure 1). Liver cells do not stain in wt as well as in KO mice while cells of the exocrine pancreas are stained in wt as well as in KO mice.

Although hyperacute rejection can be prevented in transplantation with organs from α1,3-galactosyltransferase KO, the grafts are lost after some months with signs of thrombotic microangiopathy. This could be due to the action of non-Gal antibodies that aim at proteins or carbohydrates in the transplanted organs from the GalT-KO animal [13]. The presence of non-xenoreactive antigens might also explain why GS1 B4 stains the exocrine pancreatic cells of both mouse strains. GS1 B4 is widely used as a probe for α-D-Galactosyl end groups. It has thus been shown that the isolectin detects not only the xenoreactive Galα1-3Galβ1-4GlcNAc carbohydrate but also the P blood group antigens P^k^: Galα1-4Galβ1-4Glcβ- and P_I_: Galα1-4Galβ1-4GlcNAcβ [14] and galabiose (Galα1-4Gal) which is an epithelial surface cell receptor for Shiga like toxins. GS1 B4 also exhibits broad specificity for blood group B variants and can react with type 1 [Galα1-3(Fucα1-2)Galβ1-3GlcNAc], type 2 [Galα1-3(Fucα1-2)Galβ1-4GlcNAc] and their difucosylated variants [15]. Although the xenoreactive α-Gal epitope seems to be a blood group B epitope that lacks the fucose residue it is important to realize that the two determinants are biosynthesized by two distinct α-1,3galactosyltransferases and are thus genetically unrelated [16]. It is therefore a possibility that the staining of exocrine cells in both strains reflects the presence of a glycan with a α-D-Galactosyl end group other than the xenoreactive carbohydrate.

In non-primate mammals anti-Gal antibodies may bind, not only to endothelial cells but also to epithelial cells, mesenchymal cells and extrcellular matrix glycoproteins [17]. Terminal Galα1-3Gal is thus present in both glycoprotein- and glycolipid bound forms [18] and Everett et al. [19] measured that on endothelial cells 13 percent of Galα1-3Gal are present on glycolipid and 87 percent on glycoprotein. In future modifications of immunosuppressive therapy and strategies to induce tolerance, it would be important to reveal if targeted disruption of the α1,3-galactosyltransferase gene also affects the expression of other proteins in the genetically modified animals. Some evidence for this hypothesis is reported in the literature: The content of sialic acid is altered in the GalT-KO organs and Park et al. [12] noticed an increase of Neu5Gc content in heart, lung, liver and kidney from heterozygote GalT-KO pigs. Diswall et al. [13] found increased levels of uncapped LacNAc precursor and fucosylated H type 2 determinants in GalT-KO pig tissues. It is also reported that xenotransplantation may result in formation of antibodies directed towards a series of stress response and inflammation related proteins [20]. To evaluate whether knockout of the α1,3-galactosyltransferase gene affects the expression of other proteins, we used a proteomic approach to analyze two different organs, liver and pancreas from wt as well as KO mice. These organs vary with respect to the expression of the α-Gal epitope, as it is present on the endothelial cells in the liver while absent on the endothelial cells in the pancreas. Indeed, we found several proteins as well as fragments in both organs with changed expression levels. Thus, it seems that protein turnover for some proteins is greatly influenced in the KO mice. The reason for these changes is unknown at present. Only few of the identified proteins contain N-glycosylation sites so it is unlikely that the presence or absence of the α-Gal epitope per se do play any role in the expression changes seen of proteins as well as their fragments.

### Conclusion

We have analysed two different tissues of α1,3-galactosyltransferase KO mice and revealed that deletion of this gene that directly affects the expression of the α-Gal epitope affects a number of proteins with a wide variety of functions. Thus, it seems that protein turnover, i.e., synthesis and/or degradation, for some proteins is greatly influenced in tissues of the KO mice. 

## References

[1] Chen ZC, Radic MZ, Galili U (2000) Genes coding evolutionary novel anti-carbohydrate antibodies: studies on anti-Gal production in α1,3galactosyltransferase knock out mice. Mol Immunol 37: 455-466. doi:10.1016/S0161-5890(00)00064-X. PubMed: 11090880. 11090880

[2] Breimer ME (2011) Gal/non-Gal antigens in pig tissues and human non-Gal antibodies in the GalT-KO era. Xenotransplantation 18: 215-228. doi:10.1111/j.1399-3089.2011.00644.x. PubMed: 21848538. 21848538

[3] Schmidt DO, Buschmann H-G, Hammer C (2003) Blood groups in animals. Lengerich, Germany: Pabst Science Publishers.

[4] Kirkeby S, Mikkelsen HB (2008) Distribution of the αGal- and the non-αGal T-antigens in the pig kidney: potential targets for rejection in pig-to-man xenotransplantation. Immunol Cell Biol 86: 363-371. doi:10.1038/icb.2008.1. PubMed: 18301385. 18301385

[5] Bartke A (2008) New findings in gene knockout, mutant and transgenic mice. Exp Gerontol 43: 11-14. doi:10.1016/j.exger.2007.10.009. PubMed: 18053667. 18053667

[6] Welle S, Cardillo A, Zanche M, Tawil R (2009) Skeletal muscle gene expression after myostatin knockout in mature mice. Physiol Genomics 38: 342-350. doi:10.1152/physiolgenomics.00054.2009. PubMed: 19509079. 19509079 PMC3774565

[7] Tearle RG, Tange MJ, Zannettino ZL, Katerelos M, Shinkel TA et al. (1996) The α-1,3-galactosyltransferase knockout mouse. Implications for xenotransplantation. Transplantation 61: 13-19. doi:10.1097/00007890-199601150-00004. PubMed: 8560551. 8560551

[8] Mandal N, Lewis GP, Fisher SK, Heegaard S, Prause JU et al. (2011) Protein changes in the retina following experimental retinal detachment in rabbits. Mol Vis 17: 2634-2648. PubMed: 22065916. 22065916 PMC3209431

[9] Mortz E, Krogh TN, Vorum H, Görg A (2001) Improved silver staining protocols for high sensitivity protein identification using matrix-assisted laser desorption/ionization-time of flight analysis. Proteomics 1: 1359-1363. doi:10.1002/1615-9861(200111)1:11. PubMed: 11922595. 11922595

[10] Perkins DN, Pappin DJ, Creasy DM, Cottrell JS (1999) Probability-based protein identification by searching sequence databases using mass spectrometry data. Electrophoresis 20: 3551-3567. doi:10.1002/(SICI)1522-2683(19991201)20:18. PubMed: 10612281. 10612281

[11] Kirkeby S, Hansen AK, d'Apice A, Moe D (2006) The galactophilic lectin (PA-IL, gene LecA) from Pseudomonas aeruginosa. Its binding requirements and the localization of lectin receptors in various mouse tissues. Microb Pathog 40: 191-197. doi:10.1016/j.micpath.2006.01.006. PubMed: 16542817. 16542817

[12] Park JY, Park MR, Bui HT, Kwon DN, Kang MH et al. (2012) α1,3-galactosyltransferase deficiency in germ-free miniature pigs increases N-glycolylneuraminic acids as the xenoantigenic determinant in pig-human xenotransplantation. Cell Reprogram 14: 353-363. PubMed: 22775484. 22775484 10.1089/cell.2011.0083

[13] Diswall M, Angström J, Karlsson H, Phelps CJ, Ayares D et al. (2010) Structural characterization of α1,3-galactosyltransferase knockout pig heart and kidney glycolipids and their reactivity with human and baboon antibodies. Xenotransplantation 17: 48-60. doi:10.1111/j.1399-3089.2009.00564.x. PubMed: 20149188. 20149188

[14] Kirkeby S, Moe D, Cläesson MH (1998) Galα1-->4Gal-glycans are expressed on myofibrillar associated proteins. Cell Tissue Res 293: 285-291. doi:10.1007/s004410051120. PubMed: 9662651. 9662651

[15] Ito N, Nagaike C, Morimura Y, Hatake H (1997) Estimation and comparison of the contents of blood group B antigens in selected human tissues by microphotometric quantification of Griffonia simplicifolia agglutinin I-B4 staining with or without prior α-galactosidase digestion. Histol Histopathol 12: 415-424. PubMed: 9151130. 9151130

[16] Rydberg L, Holgersson J, Samuelsson BE, Breimer ME (1999) α-Gal epitopes in animal tissue glycoproteins and glycolipids. Subcell Biochem 32: 107-125. PubMed: 10391993. 10391993 10.1007/978-1-4615-4771-6_5

[17] Maruyama S, Cantu E 3rd, Galili U, D'Agati V, Godman G et al. (2000) α-galactosyl epitopes on glycoproteins of porcine renal extracellular matrix. Kidney Int 57: 655-663. doi:10.1046/j.1523-1755.2000.t01-1-00887.x. PubMed: 10652044. 10652044

[18] Strokan V, Mölne J, Svalander CT, Breimer ME (1998) Heterogeneous expression of Gal α1-3Gal xenoantigen in pig kidney: a lectin and immunogold electron microscopic study. Transplantation 66: 1495-1503. doi:10.1097/00007890-199812150-00013. PubMed: 9869091. 9869091

[19] Everett ML, Lin SS, Worrell SS, Platt JL, Parker W (2003) The footprint of antibody bound to pig cells: evidence of complex surface topology. Biochem Biophys Res Commun 301: 751-757. doi:10.1016/S0006-291X(03)00043-3. PubMed: 12565844. 12565844

[20] Byrne GW, Stalboerger PG, Davila E, Heppelmann CJ, Gazi MH et al. (2008) Proteomic identification of non-Gal antibody targets after pig-to-primate cardiac xenotransplantation. Xenotransplantation 15: 268-276. doi:10.1111/j.1399-3089.2008.00480.x. PubMed: 18957049. 18957049 PMC2586876

